# Calcitriol Supplementation Ameliorates Microvascular Endothelial Dysfunction in Vitamin D-Deficient Diabetic Rats by Upregulating the Vascular eNOS Protein Expression and Reducing Oxidative Stress

**DOI:** 10.1155/2021/3109294

**Published:** 2021-02-02

**Authors:** Chee Lee Wee, Siti Safiah Mokhtar, Kirnpal Kaur Banga Singh, Sahran Yahaya, Susan Wai Sum Leung, Aida Hanum Ghulam Rasool

**Affiliations:** ^1^Department of Pharmacology, School of Medical Sciences, Universiti Sains Malaysia, 16150 Kota Bharu, Kelantan, Malaysia; ^2^Department of Microbiology and Parasitology, School of Medical Sciences, Universiti Sains Malaysia, 16150 Kota Bharu, Kelantan, Malaysia; ^3^Department of Orthopaedics, School of Medical Sciences, Universiti Sains Malaysia, 16150 Kota Bharu, Kelantan, Malaysia; ^4^Department of Pharmacology and Pharmacy, Li Ka Shing Faculty of Medicine, The University of Hong Kong, Hong Kong, China

## Abstract

Diabetes mellitus contributes to macro- and microvascular complications, leading to adverse cardiovascular events. This study examined the effects of vitamin D deficiency on the vascular function and tissue oxidative status in the microcirculation of diabetic rats and to determine whether these effects can be reversed with calcitriol (active vitamin D metabolite) supplementation. Streptozotocin-induced diabetic rats were fed for 10 weeks with control diet (DC) or vitamin D-deficient diet without (DD) or with oral calcitriol supplementation (0.15 *μ*g/kg) in the last four weeks (DDS) (10 rats each group). A nondiabetic rat group that received control diet was also included (NR). After 10 weeks, rats were sacrificed; mesenteric arterial rings with and without endothelium were studied using wire myograph. Western blotting of the mesenteric arterial tissue was performed to determine the protein expression of endothelial nitric oxide synthase (eNOS) enzyme. Antioxidant enzyme superoxide dismutase (SOD) activity and oxidative stress marker malondialdehyde (MDA) levels in the mesenteric arterial tissue were also measured. The DC group had significantly lower acetylcholine-induced relaxation and augmented endothelium-dependent contraction, with reduced eNOS expression, compared to NR rats. In mesenteric arteries of DD, acetylcholine-induced endothelium-dependent and sodium nitroprusside-induced endothelium-independent relaxations were lower than those in DC. Calcitriol supplementation in DDS restored endothelium-dependent relaxation. Mesenteric artery endothelium-dependent contraction of DD was greater than DC; it was not affected by calcitriol supplementation. The eNOS protein expression and SOD activity were significantly lower while MDA levels were greater in DD compared to DC; these effects were not observed in DDS that received calcitriol supplementation. In conclusion, vitamin D deficiency causes eNOS downregulation and oxidative stress, thereby impairing the vascular function and posing an additional risk for microvascular complications in diabetes. Calcitriol supplementation to diabetics with vitamin D deficiency could potentially be useful in the management of or as an adjunct to diabetes-related cardiovascular complications.

## 1. Introduction

Diabetes mellitus (DM), which is characterized by chronic hyperglycemia resulting from defective insulin secretion and/or insulin action [1], is a major growing threat to public health. This disease ranks as the world top ten leading causes of death in year 2019 and constitutes 11.3% of global all-cause mortality, causing 4.2 million deaths globally in year 2019 [2]. DM is gaining global concern for its epidemic proportion with an increasing global prevalence of 463 million (9.3%) in year 2019 and is estimated to rise beyond 700 million (10.6%) in year 2045 [2].

DM poses significant risks of macro- and microvascular complications, which eventually lead to a higher risk of developing adverse cardiovascular events. Cardiovascular diseases (CVD) are the major contributor to morbidity and mortality in diabetics [3]. Hence, it is critical to recognise and treat this devastating disease early to delay or even prevent serious diabetes-related secondary cardiovascular complications.

Low vitamin D levels have been reported as one of the risk factors in the development of DM and CVD [4–6]. Lower vitamin D levels are associated with a 40% higher risk of developing DM in women [7] and a 50% increased risk of developing CVD when compared to subjects with optimal vitamin D levels [8]. Regrettably, vitamin D insufficiency and deficiency are a common public health issue, not only in Western societies with seasonal changes but also in tropical countries [9–12]. On top of that, the prevalence appears to be more pronounced in the diabetic population [7, 13].

Aside from its well-known classical roles in calcium and phosphate homeostasis and in bone metabolism, accumulating evidence reported that vitamin D is a steroidal hormone that plays a pivotal role through vitamin D receptors (VDR) in vascular homeostasis and in the modulation of the endothelial function [14]. Low vitamin D levels have been discovered to be closely associated with endothelial dysfunction in both normal and diabetic populations [15, 16]. Endothelial dysfunction, which is characterized by impaired endothelium-dependent relaxation, is an independent precursor of early atherosclerotic development and prognosis of CVD [17, 18]. The presence of endothelial dysfunction in the microcirculation is reported to serve as an important prognostic marker for early manifestation of atherosclerotic risk, before the onset of atherosclerosis [19].

In addition, vitamin D deficiency has also been reported to be closely associated with enhanced vascular oxidative stress in both normal and diabetic models [20–22]. Augmented oxidative stress due to the disruption in the homeostasis between the enhanced free radical-generating process and/or the impaired capacity of the antioxidant defense systems to scavenge the excess free radicals is also one of the key contributors in the pathogenesis of diabetes-related secondary complications [23].

Considering the adverse impacts of low vitamin D levels in the development of cardiovascular complications, particularly in the diabetic population, there is an urgency to study the possible underlying pathology of low vitamin D levels in the development of diabetes-related cardiovascular complications. To our best knowledge, prior vascular studies on vitamin D deficiency are mainly focused on the diabetes macrocirculation, while the effects of vitamin D deficiency on the vascular function in the diabetes microcirculation remain poorly understood. In view of this knowledge gap, the present study was designed to investigate the effects of vitamin D deficiency on the microvascular function, which consists of endothelial and smooth muscle responses, in streptozotocin-induced diabetic rats. This study also assessed whether or not the vascular functional abnormalities attributed to vitamin D deficiency involved the alterations in the protein expression of endothelial nitric oxide synthase (eNOS), the enzyme responsible for producing the major endothelium-derived vasodilator nitric oxide (NO), in the microvascular tissue of diabetic rats with vitamin D deficiency.

To date, there seems to be a lack of data reporting on the effects of vitamin D deficiency on oxidative stress levels in the microcirculation of diabetics as a possible mechanism accounting for the microvascular dysfunction. Hence, this study also determined the levels of oxidative stress parameters which include superoxide dismutase (SOD; an antioxidant enzyme) and malondialdehyde (MDA; the product of lipid peroxidation) in the microvascular tissue of diabetic rats with different vitamin D status. In addition, the potential of calcitriol (the active metabolite of vitamin D) supplementation to reverse or alleviate the adverse effects attributed to low vitamin D levels on the microvascular function and microvascular tissue oxidative stress levels was examined.

## 2. Materials and Methods

### 2.1. Materials

A standard chow diet (AIN93G) and a modified vitamin D-deficient diet (SF03-009) were purchased from Specialty Feeds Pty Ltd. (Glen Forrest, Western Australia). The standard diet contains 1000 IU/kg of vitamin D_3_. Both diets also contained 0.47% calcium and 0.35% phosphorus. Acetylcholine hydrochloride, calcium ionophore, sodium nitroprusside, phenylephrine hydrochloride, and streptozotocin (STZ) were purchased from Sigma Chemical Co. (St. Louis, MO, USA). Salbutamol hemisulfate was purchased from Tocris Bioscience (Bristol, UK). _L_-NAME hydrochloride was obtained from Cayman Chemicals (Ann Arbor, MI, USA). Calcitriol (Rocaltrol) was purchased from Roche (Switzerland). Primary antibody against eNOS (AB76198), mouse monoclonal antibody to *β*-actin (AB8226), and horseradish peroxidase- (HRP-) conjugated secondary antibody (AB6721) were purchased from Abcam (Cambridge, UK).

### 2.2. Animals and Experimental Protocols

The approval for experimental protocols used in this study was obtained from the Animal Ethics Committee of Universiti Sains Malaysia (AECUSM) [No. USM/Animal Ethics Approval/2016/(98)(711)]. 40 male Sprague-Dawley rats aged 8-9 weeks and weighing between 250–350 g were used. The rats were acclimatised for 1-2 weeks in Animal Research and Service Centre (ARASC), Universiti Sains Malaysia. 30 rats were randomly chosen to be induced with diabetes while 10 rats formed the nondiabetic control group. After an overnight fast, type 1 diabetes was induced with a single intraperitoneal injection of freshly prepared STZ (50 mg/kg) dissolved in citrate buffer (0.01 M, pH 4.5) while the nondiabetic control group received vehicle. After 72 hours of diabetes induction and an overnight fast, blood samples were obtained via the tail prick method, and the glucose concentration was measured using a one-touch glucometer (Accu-check, Roche Diagnostics, Indianapolis, IN, USA). The induction of diabetes was considered successful when the fasting blood glucose (FBG) levels were at least 16.0 mmol/L [24]. Body weight and FBG levels measured at the day of successful diabetes induction were recorded as the baseline value.

Diabetic rats were then randomly divided into three groups as follows: diabetic rats that received a 10-week standard chow diet (group DC)—the diabetic control group, diabetic rats that received a 10-week vitamin D-deficient diet (group DD)—the vitamin D-deficient diabetic group, and diabetic rats that received a 10-week vitamin D-deficient diet, with 4-week oral calcitriol supplementation (0.15 *μ*g/kg), starting from week 7 of diabetes induction until the end of the study period (group DDS)—the treated group. Nondiabetic rats received a 10-week standard chow diet (group NR). Rats were placed in cages at ARASC with the temperature maintained at 25°C with a 12-hour light/dark cycle. All rats had access to tap water ad libitum throughout the experiments. Food pellet was given regularly at 25-30 g daily. At the end of 10 weeks after diabetes induction, rats were anaesthetised with an intraperitoneal injection of pentobarbitone sodium (70 mg/kg) and exsanguinated. Body weight and FBG levels measured on the day of sacrifice were taken as the final value for the parameters. Blood samples were taken from the left ventricle via the cardiac puncture for the measurements of serum 25-hydroxyvitamin D (25(OH)D) levels using the 25-hydroxy vitamin D enzyme immunoassay (#AC-57F1, IDS, Tyne & Wear, UK) and calcium levels by sending to the certified laboratory (BP Lab, Malaysia).

### 2.3. Microvascular Function Studies

Upon sacrifice, the mesenteric vascular bed was isolated and immediately placed on a petri dish with ice-cold and oxygenated modified Krebs solution (119 mM NaCl, 4.7 mM KCl, 1.2 mM MgSO_4_, 1.2 mM KH_2_PO_4_, 25 mM NaHCO_3_, 5.5 mM D-glucose, and 2.5 mM CaCl_2_). The small mesenteric arteries (first-order branch of the superior mesenteric artery) were isolated from the mesenteric vascular bed. The arteries were cleared of excessive fat and surrounding adhering tissue and cut into 2 mm-long ring segments under a dissecting microscope. Care was taken during the dissection to prevent any damage to the delicate endothelial layer. In some preparations, the endothelial cells were removed by rubbing the intimal surface of the arteries with human hair.

Ring segments were mounted in the organ chamber of wire myograph (model 410A, Danish Myo Technology, Aarhus, Denmark) filled with modified Krebs solution. The bathing solution was maintained at 37°C and continuously aerated with carbogen (95% O_2_ and 5% CO_2_; pH 7.4). The rings were equilibrated for 30 minutes before being normalized to set the vessels to its standard initial conditions, followed by 60 minutes equilibration before proceeding for isometric tension measurements. Afterwards, the rings were exposed twice to potassium chloride (60 mM) to obtain a reference contraction and to ensure smooth muscle viability. Following that, rings were tested for the integrity of the endothelium by precontracting with phenylephrine (10^−5^ M). When a steady contraction to phenylephrine was obtained, acetylcholine (10^−5^ M) was added to assess the presence or absence of functional endothelial cells.

Endothelium-dependent relaxation and contraction were studied in rings with endothelium. For endothelium-dependent relaxation, rings with endothelium were precontracted with phenylephrine (10^−5^ M) followed by cumulative addition of acetylcholine (10^−8^ to 10^−4^ M). Endothelium-dependent contraction was studied using cumulative concentrations of calcium ionophore (10^−8^ to 10^−4^ M) in the quiescent rings with endothelium preincubated with _L_-NAME (10^−4^ M) (eNOS inhibitor) for 30 minutes. Incubating the vessels with _L_-NAME inhibits the catalytic activity of eNOS for NO production, potentiating vasoconstriction. Endothelium-independent relaxation and contraction were determined in rings without endothelium. For endothelium-independent relaxation, rings without endothelium were precontracted with phenylephrine (10^−5^ M) followed by cumulative addition of sodium nitroprusside or salbutamol (10^−8^ to 10^−4^ M). Endothelium-independent contraction was determined using cumulative concentrations of phenylephrine (10^−8^ to 10^−4^ M) in the quiescent rings without endothelium.

### 2.4. Oxidative Stress Analysis

Mesenteric arteries cleaned of excess fat and connective tissues were finely chopped in a microcentrifuge tube filled with prechilled lysis buffer (NaCl 150 mM, Tris 50 mM, Triton-X 1%, sodium dioxycholate 10%, sodium dodecyl sulfate (SDS) 0.1%, ethylenediaminetetraacetic acid (1 mM), and a protease inhibitor cocktail 0.05% (Sigma Chemical Co., St. Louis, MO, USA) on ice using dissecting scissors. The tissue lysate was then incubated at 4°C for 10 minutes, followed by centrifugation at 12,000× *g* at 4°C. SOD activities in the tissue samples were quantitatively determined by using EnzyChrom™ Superoxide Dismutase Assay Kit (ESOD-100, BioAssay Systems, USA), while MDA concentrations in vascular tissue samples were measured using the NWLSS™ Malondialdehyde Assay (NWK-MDA01, Northwest Life Science Specialty, USA), following the manufacturer's instruction.

### 2.5. Western Blot Analysis

The supernatant of the mesenteric arterial tissue was collected. Protein concentrations were determined using the Bradford assay. In all immunoblot experiments, the same mass of protein (30 *μ*g) was loaded in each lane of 10% SDS-polyacrylamide gel. After electrophoresis, proteins were electrotransferred to Immobilon-P polyvinylidene difluoride membranes (Merck, Germany) and incubated for 16 hours at 4°C with primary antibodies against eNOS (1 : 1000 Abcam, Cambridge, UK). To normalize the amount of protein, *β*-actin was used as a loading control (1 : 5000, Abcam, Cambridge, UK). The membranes were then incubated in HRP-conjugated polyclonal secondary antibody (1 : 1000 for eNOS and 1 : 2000 for *β*-actin; Abcam) in blocking buffer for one hour at room temperature. The membranes were incubated with chemiluminescent HRP substrate (Nacalai Tesque, Japan) for 5 minutes and exposed to Fusion Molecular Imager (Vilber Lourmat, France). The intensity of protein bands representing the amount of protein was measured with Image J software version 1.8.0_112. The relative protein presence of eNOS was expressed as the percentage of the total amount of protein (indicated by the intensity of the protein band for *β*-actin) in the same animal sample [25].

### 2.6. Statistical Analysis

Continuous variables are expressed as mean (standard deviation) for Gaussian distributions or median (interquartile range) for non-Gaussian distributions. Continuous variables were compared using a one-way analysis of variance (ANOVA) or Kruskal-Wallis test, as appropriate. All analyses were performed using IBM Statistical Package for the Social Sciences (SPSS) software version 24.0 (Armonk, NY, USA). A *p* value of <0.05 was considered as statistically significant.

## 3. Results

### 3.1. Basic and Laboratory Parameters

Nondiabetic rats (NR) had similar baseline body weight as the diabetic groups at the beginning of the study. However, NR had significantly higher body weight at the end of the study compared to diabetic groups. The baseline and final body weights were similar in all diabetic groups. Similarly, baseline and final FBG levels were also comparable among the diabetic groups. The levels of serum 25(OH)D were comparable between groups NR and DC. Serum 25(OH)D levels were significantly lower in DD and DDS compared to DC. Supplementation with calcitriol for 4 weeks in DDS did not improve vitamin D levels when compared to DD and remained significantly lower than DC. There was no significant difference in serum calcium levels between all study groups (Table 1).

### 3.2. Microvascular Function Studies

**Figure 1:**
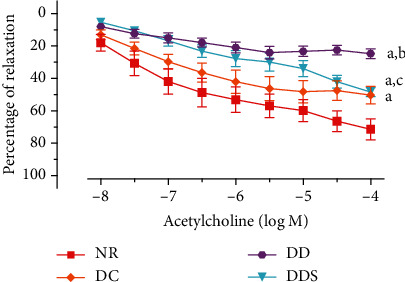
Concentration-relaxation curves of the endothelium-dependent vasodilator acetylcholine in mesenteric arteries, contracted with phenylephrine (10^−5^ M) of experimental rats. Data are presented as mean ± SD, *n* = 10 rats, ^a^*p* < 0.05 vs NR, ^b^*p* < 0.05 vs DC, ^c^*p* < 0.05 vs DD [NR: nondiabetic rats with control diet; DC: diabetic rats with control diet; DD: diabetic rats with vitamin D-deficient diet; DDS: diabetic rats with vitamin D-deficient diet+calcitriol supplementation].

**Figure 2:**
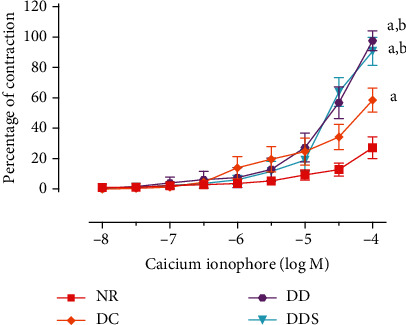
Concentration-contraction curves of calcium ionophore, in the presence of _L_-NAME (endothelial nitric oxide synthase inhibitor, 10^−4^ M), in quiescent mesenteric arteries of experimental rats. Data are presented as mean ± SD, *n* = 10 rats, ^a^*p* < 0.05 vs NR, ^b^*p* < 0.05 vs DC [NR: nondiabetic rats with control diet; DC: diabetic rats with control diet; DD: diabetic rats with vitamin D-deficient diet; DDS: diabetic rats with vitamin D-deficient diet + calcitriol supplementation].

**Figure 3:**
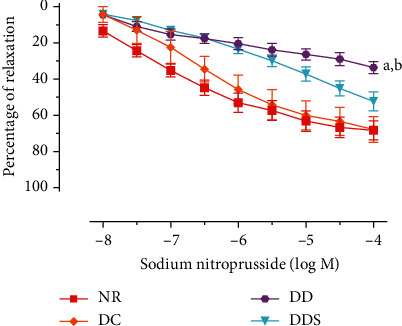
Concentration-relaxation curves of sodium nitroprusside in mesenteric arteries without endothelium, contracted with phenylephrine (10^−5^ M) of experimental rats. Data are presented as mean ± SD, *n* = 10 rats, ^a^*p* < 0.05 vs NR, ^b^*p* < 0.05 vs DC [NR: nondiabetic rats with control diet; DC: diabetic rats with control diet; DD: diabetic rats with vitamin D-deficient diet; DDS: diabetic rats with vitamin D-deficient diet+calcitriol supplementation].

**Figure 4:**
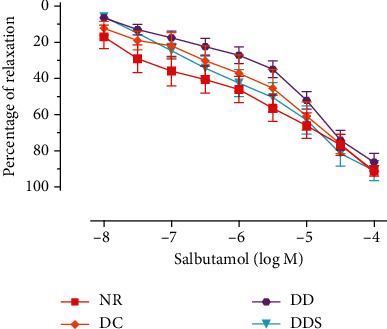
Concentration-relaxation curves of salbutamol in mesenteric arteries without endothelium, contracted with phenylephrine (10^−5^ M) of experimental rats. Data are presented as mean ± SD, *n* = 10 rats, and there was no significant difference between study groups [NR: nondiabetic rats with control diet; DC: diabetic rats with control diet; DD: diabetic rats with vitamin D-deficient diet; DDS: diabetic rats with vitamin D-deficient diet + calcitriol supplementation].

**Figure 5:**
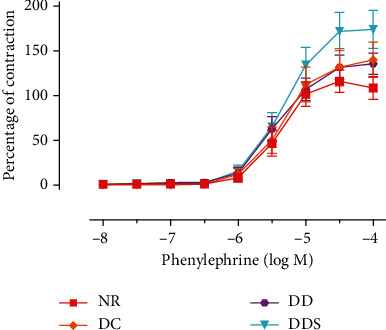
Concentration-contraction curves of phenylephrine in quiescent mesenteric arteries without endothelium of experimental rats. Data are presented as mean ± SD, *n* = 10 rats, and there was no significant difference between study groups [NR: nondiabetic rats with control diet; DC: diabetic rats with control diet; DD: diabetic rats with vitamin D-deficient diet; DDS: diabetic rats with vitamin D-deficient diet + calcitriol supplementation].

Endothelium-dependent relaxation induced by acetylcholine (ACh) was significantly attenuated in mesenteric arteries of DC compared to NR (Figure 1; Table 2). The maximal endothelium-dependent relaxation was also further significantly reduced in mesenteric arteries of DD compared to DC (Figure 1; Table 2). Calcitriol supplementation for 4 weeks in DDS showed a significant improvement in ACh-induced maximal relaxation compared to DD; the maximal relaxation was comparable to group DC (Figure 1; Table 2). Endothelium-dependent contraction induced by calcium ionophore (CaI) was significantly augmented in mesenteric arteries of DC compared to NR (Figure 2; Table 2). The maximal endothelium-dependent contraction was also further significantly augmented in mesenteric arteries of DD compared to DC (Figure 2; Table 2). Calcitriol supplementation for 4 weeks in DDS did not cause significant reduction in endothelium-dependent contraction compared to DD; the maximal contraction remained significantly higher compared to group DC (Figure 2; Table 2). The maximal relaxation to sodium nitroprusside (SNP) in mesenteric arteries of DC showed no significant difference compared to NR (Figure 3; Table 2). However, the mesenteric arteries of DD had significantly lower maximal relaxation to SNP compared to DC (Figure 3; Table 2). There was no significant difference in SNP-induced maximal relaxation in the DDS group that received calcitriol supplementation compared to DD (Figure 3; Table 2). There were no significant differences in the responses of vascular smooth muscle cells (VSMC) in the mesenteric arteries to salbutamol (SB) and phenylephrine (PE) in any of the experimental groups (Figures 4 and 5; Table 2).

### 3.3. Tissue Oxidative Stress Analysis

**Figure 6:**
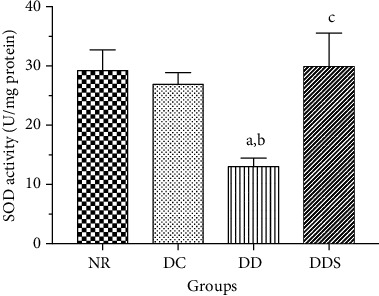
Superoxide dismutase (SOD) activity in the mesenteric arterial tissue of experimental rats. Data are presented as mean ± SD, *n* = 10 rats, ^a^*p* < 0.05 vs NR, ^b^*p* < 0.05 vs DC, ^c^*p* < 0.05 vs DD [NR: nondiabetic rats with control diet; DC: diabetic rats with control diet; DD: diabetic rats with vitamin D-deficient diet; DDS: diabetic rats with vitamin D-deficient diet + calcitriol supplementation].

There was no significant difference in the SOD activity between groups NR and DC (Figure 6). Rats in the DD group had significantly lower SOD activity compared to DC (Figure 6). However, calcitriol supplementation significantly increased the SOD activity in DDS compared to DD, and the SOD activity was comparable to DC (Figure 6).

**Figure 7:**
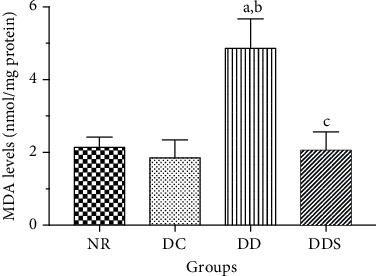
Malondialdehyde (MDA) levels in the mesenteric arterial tissue of experimental rats. Data are presented as mean ± SD, *n* = 10 rats, ^a^*p* < 0.05 vs NR, ^b^*p* < 0.05 vs DC, ^c^*p* < 0.05 vs DD. [NR: nondiabetic rats with control diet; DC: diabetic rats with control diet; DD: diabetic rats with vitamin D-deficient diet; DDS: diabetic rats with vitamin D-deficient diet+calcitriol supplementation].

Similarly, there was no significant difference in MDA levels between groups NR and DC (Figure 7). Rats in the DD group had significantly higher MDA levels compared to DC (Figure 7). Calcitriol supplementation significantly reduced MDA levels in DDS when compared to DD, and the MDA levels were comparable to DC (Figure 7).

### 3.4. Western Blot Analysis

**Figure 8:**
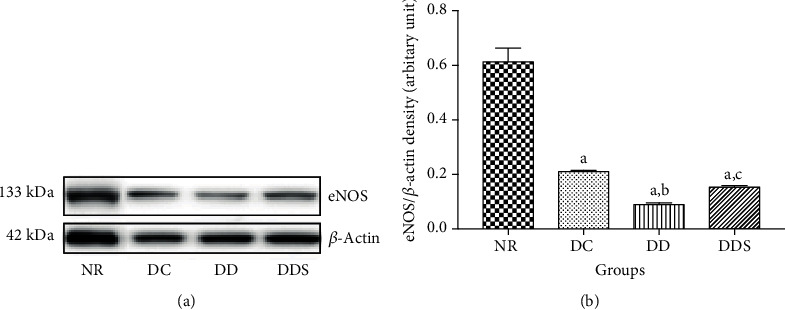
(a) Representative Western blot results demonstrated the endothelial nitric oxide synthase (eNOS) protein expression in mesenteric arteries of experimental rats. (b) Graphical presentation of the data normalized to beta-actin. Data are presented as mean ± SD (*n* = 10). ^a^*p* < 0.05 vs NR, ^b^*p* < 0.05 vs DC, ^c^*p* < 0.05 vs DD [NR: nondiabetic rats with control diet; DC: diabetic rats with control diet; DD: diabetic rats with vitamin D-deficient diet; DDS: diabetic rats with vitamin D-deficient diet+calcitriol supplementation].

The expression levels of the eNOS protein were significantly lower in the mesenteric arteries of DC compared to NR (Figure 8). The expression levels of the eNOS protein were further significantly reduced in the mesenteric arteries of DD compared to DC (Figure 8). Calcitriol supplementation increased the expression levels of the eNOS protein in the mesenteric arteries of DDS compared to DD (Figure 8).

## 4. Discussion

The present study showed that in small mesenteric arteries of diabetic rats, endothelium-dependent relaxation was significantly impaired, whilst contraction was augmented, compared to nondiabetic rats. The endothelial function in the microcirculation of diabetic rats was further worsened by vitamin D deficiency. Moreover, smooth muscle relaxations to sodium nitroprusside were attenuated, but those to salbutamol were preserved in small mesenteric arteries of diabetic rats with vitamin D deficiency. The changes in the microvascular function in vitamin D-deficient diabetic rats, compared to diabetic controls, are associated with higher oxidative stress, as shown by increased MDA levels and reduced SOD activity, and with reduced protein expression of eNOS. Calcitriol supplementation to vitamin D-deficient diabetic rats improved endothelium-dependent relaxation, enhanced the mesenteric arterial tissue SOD activity and protein expression of eNOS, and reduced MDA levels.

The delicate balance between endothelium-dependent relaxation and contraction maintains vascular homeostasis and modulates vascular tone [26]. Endothelial dysfunction is characterized by the imbalance in the production and action between endothelium-derived relaxing and contracting factors [27]. In the present study, endothelial dysfunction is worsened in the microcirculation of vitamin D-deficient diabetic rats, as shown by diminished endothelium-dependent relaxation and augmented contraction. This may be due to the reduced protein expression of eNOS and enhanced oxidative stress levels attributed to vitamin D deficiency.

eNOS catalyzes the production of NO from _L_-arginine and molecular oxygen in endothelial cells [28]. NO is the major potent vasodilator that modulates endothelium-dependent relaxation in most vessels [27, 29]. Hyperglycemia has been associated with reduced eNOS protein expression in diabetes [30], where decreased protein levels of eNOS were reported in cultured human coronary arterial and aortic endothelial cells that were incubated in media with elevated glucose concentrations [30, 31]. Lower eNOS protein expression has also been reported in tail arteries of STZ-induced diabetic rats [25]. The reduced eNOS expression was also observed in the aorta of VDR mutant mice [32, 33]. Vitamin D was reported to be involved in enhancing the transcription of eNOS and nongenomic activation of eNOS, to promote NO production in endothelial cells. The reduced protein expression of eNOS leads to reduced endothelial production of NO, which is a major vasodilator for the regulation of vascular tone, leading to the impairment in endothelium-dependent relaxation.

Augmented oxidative stress levels due to the imbalance between excess production of reactive oxygen species (ROS) and reduced antioxidant activities [34] play a dominant role in the development of diabetes-related vascular complications [35]. In the present study, augmented microvascular tissue oxidative stress was exhibited by enhanced MDA levels and reduced SOD activity in vitamin D-deficient diabetic rats compared to diabetic controls.

MDA, which is one of the byproducts of polyunsaturated fatty acid peroxidation in the cells, is the most frequently used biomarker of oxidative stress in CVD [36]. The measurement of MDA levels is reliably documented as the gold standard [37] to indicate the levels of cellular peroxidative injury attributable to augmented ROS levels; higher MDA levels represent higher levels of ROS that ultimately lead to a higher degree of lipid peroxidation. Vitamin D-deficient diabetic rats showed significantly increased MDA levels compared to diabetic controls. Chronic hyperglycemia in diabetes could serve as an additional source of mitochondrial ROS overproduction by both enzymatic and nonenzymatic pathways, thereby augmenting oxidative stress [38]. Besides that, vitamin D deficiency impairs mitochondrial respiratory function (excessive respiration leads to elevated ROS production), upregulates the gene expression of NADPH oxidase (the key producer of ROS), and downregulates cellular antioxidants expression, leading to the elevated formation of free radicals and ROS [39]. An enhanced increase in free radicals diminishes the antioxidant activities and depletes antioxidant enzymes, further propagating the free radical chain reactions that subsequently lead to peroxidative injury. Increased MDA levels have been reported in diabetic animal models [40–42].

SOD is a major enzyme of the antioxidant defense system to counteract oxidative stress and subsequently reduce the risk of cellular injury [43]. SOD catalyzes the dismutation of superoxide anion to hydrogen peroxide and molecular oxygen [44]. SOD levels may be low and diminished due to its depletion, presumably as a compensatory response of its antioxidant defense activity against excess ROS over time [45–47]. Reduced SOD levels and activity have been reported in vitamin D-deficient subjects [20] and in diabetic animal models [40–42].

In addition, augmented oxidative stress reduces NO bioavailability, leading to endothelial dysfunction [48]. Increased superoxide anion production induced by vitamin D deficiency and hyperglycemia impairs the catalytic activity of eNOS by promoting the dissociation of eNOS dimer to its monomer [49]. The uncoupled and hence dysfunctional eNOS is shifted to produce superoxide anion, thus compromising cellular NO generation by eNOS [50, 51]; this accounts for the impairment in NO-mediated relaxation in mesenteric arteries of vitamin D-deficient diabetic rats. Furthermore, excess superoxide anion scavenges NO produced by eNOS avidly to form peroxynitrite: the resultant-reduced bioavailability of NO further contributed to the attenuated NO-mediated relaxation in diabetic animals. Reduced endothelium-dependent relaxation has also been reported to be associated with increased oxidative stress levels [52, 53].

In certain pathological conditions such as diabetes, attenuated NO production initiates the contraction of the vascular smooth muscle [24]. The reduced bioavailability of NO induces the production and release of endothelium-derived vasoconstrictor prostanoids and endothelin-1 [54], leading to vasoconstriction which amplifies the degree of endothelial dysfunction. In the present study, vitamin D-deficient diabetic rats demonstrated markedly augmented endothelium-dependent contraction compared to diabetic controls. This is in line with a previous study that reported augmented endothelium-dependent contraction induced by calcium ionophore in femoral arteries of STZ-induced rats [24]. The overproduction of ROS as induced by vitamin D deficiency coupled with hyperglycemia might also enhance the activity of cyclooxygenase in endothelial or smooth muscle cells to release prostanoid, thus augmenting vasoconstriction [55]. Superoxide anions might effectively scavenge NO, favoring the production of cyclooxygenase products responsible for vasoconstriction (such as thromboxane A_2_) and evokes augmented endothelium-dependent vasoconstriction and endothelial dysfunction [24, 55]. An enhanced endothelium-dependent contraction in diabetes might also relate to hyperglycemia-induced augmented oxidative stress and upregulation of the cyclooxygenase-2 protein expression, as reported in human aortic endothelial cells cultured in high glucose medium [56].

Impaired NO-mediated relaxation can be a consequence of reduced production and release of NO in the endothelial cells, but can also be due to reduced sensitivity of VSMC to NO. In this study, the response of VSMC to the exogenous NO donor SNP was significantly attenuated in vitamin D-deficient diabetic rats compared to diabetic controls, thus suggesting that vitamin D deficiency also caused impairment in VSMC function. Indeed, Okon et al. (2003) showed that the sensitivity of VSMC to NO was decreased in the aorta and mesenteric internal arteries from diabetic mice [57]. Impaired endothelium-independent responses in vitamin D-deficient diabetic rats may be explained by the reduced bioavailability of NO [58], released directly from SNP, in view of the increased oxidative stress with vitamin D deficiency. The reduction in NO-mediated vascular smooth muscle relaxation could also in part be explained by the impairment in the cyclic GMP pathway, which is involved in SNP-induced vascular smooth muscle relaxation [59], such as the reduction in the expression of soluble guanylyl cyclase (the enzyme for generating cyclic GMP), the abnormality in the production of cyclic GMP, or the dysfunction of the cyclic GMP-dependent protein kinase responsible for inducing vasodilatation [60]. Since the vascular smooth muscle responses to cyclic AMP-dependent vasodilator salbutamol and to the alpha-adrenergic receptor agonist phenylephrine were not affected, the reduced SNP-induced relaxation in vitamin D-deficient diabetic rat mesenteric arteries is unlikely caused by a dysfunction of vascular smooth muscle contractile machinery.

The present finding showed that calcitriol supplementation did not restore the serum 25(OH)D levels in vitamin D-deficient diabetic rats; this is not unexpected due to the type of vitamin D analogue given to the supplemented group. Vitamin D-deficient rats were supplemented with calcitriol (1,25(OH)_2_D) instead of calcidiol (25(OH)D) in the present study. Calcitriol is the physiologically active metabolite converted from 25(OH)D in the presence of 25(OH)D-1*α*-hydroxylase during the second hydroxylation process in the kidney. Therefore, serum 25(OH)D levels remained low in the calcitriol-supplemented group; nevertheless, the serum concentrations of the active form of vitamin D, 1,25(OH)_2_D, may be sufficient to produce biological effects, as demonstrated by the differences in the mesenteric arterial function in vitamin D-deficient diabetic rats without and with calcitriol supplementation. Calcitriol was used in this study in view that it is more efficient in producing biological effects as it is the active metabolite of vitamin D_3_ compared to other vitamin D_3_ analogues such as cholecalciferol and calcidiol.

Calcitriol supplemented to vitamin D-deficient diabetic rats significantly reversed the impaired endothelium-dependent relaxation to the degree comparable to that in diabetic controls. This is likely due to the significant improvement in their oxidative status, as indicated by the reduced MDA levels and increased SOD activity. The protein expression of eNOS in the mesenteric arterial tissue of vitamin D-deficient diabetic rats with calcitriol supplementation was also upregulated compared to those without. The latter finding can be attributed to reduced oxidative stress [30] or to direct activation of VDR by calcitriol since VDR deficiency is associated with the reduced aortic eNOS expression [33]. Reduced oxidative stress, coupled with the enhanced protein expression of eNOS, thus accounts for the improvement in endothelium-dependent relaxation. However, the reduced oxidative stress levels are not associated with amelioration of endothelium-dependent contraction or improvement of vascular smooth muscle responses to NO donor. Further studies are warranted to identify the mechanism(s) underlying the effects of vitamin D deficiency on these detrimental vascular effects and to determine whether or not a longer period of calcitriol supplementation can reverse these effects.

The present study has some limitations. Firstly, the actual contribution of the eNOS monomer/dimer ratio in affecting the total eNOS expression in rats' mesenteric arterial tissues was not determined. To measure the eNOS monomer/dimer ratio, the process of electrophoresis during Western blotting needs to be conducted in cold condition. Unfortunately, we lack sufficient microvascular tissue samples and faced difficulties in conducting this additional experimental protocol under such special condition. Secondly, the microvascular NO levels were not determined in this study; however, reduced endothelium-dependent relaxation in the microcirculation is most likely due to the reduced synthesis and/or availability of NO. This is in view of NO being reported as the major endothelium-derived relaxing factor in large conduit vessels [61, 62], as well as in the microcirculation, such as tail arteries and mesenteric arteries [25, 49]. Thirdly, this study also did not measure microvascular tissue cGMP and VDR levels. The measurement of tissue levels of cGMP, by radioimmunoassay or scintillation proximity assay, is important in studies of NO-cGMP signal transduction [60]. VDR has been reported to play an important role in the regulation of vascular tone and shown to be associated with the reduced eNOS expression and function [32, 33]. Hence, future studies are recommended to address these parameters to elucidate their possible roles in diabetic vascular dysfunction with vitamin D deficiency.

## 5. Conclusion

To the best of our knowledge, this is the first study demonstrating exacerbation of microvascular dysfunction in diabetic rats with vitamin D deficiency. This is associated with the reduced vascular eNOS protein expression and augmented oxidative stress. Furthermore, this study also showed that endothelial dysfunction in vitamin D-deficient diabetics is able to be ameliorated by calcitriol supplementation, accompanied by increased vascular eNOS protein expression and improvement in oxidative status. Hence, vitamin D supplementation may be a potential adjunct treatment for diabetics, especially those with vitamin D deficiency, to prevent or ameliorate vascular dysfunction, potentially improving diabetic vasculopathy.

## Figures and Tables

**Table 1:** Body weight, fasting blood glucose (FBG) levels, serum 25-hydroxyvitamin D (25(OH)D), and calcium levels for experimental rats.

Parameters	NR	DC	DD	DDS	*p* value
Body weight (baseline), g	250.70 ± 28.39	257.80 ± 26.00	261.00 ± 27.82	233.70 ± 32.62	0.167
Body weight (final), g	442.60 ± 74.15	278.60 ± 45.28^a^	316.90 ± 53.63^a^	276.50 ± 31.58^a^	<0.001
FBG levels (baseline), mmol/L	5.98 ± 0.42	25.85 ± 4.83^a^	26.39 ± 4.45^a^	28.63 ± 4.36^a^	<0.001
FBG levels (final), mmol/L	5.79 ± 0.58	31.39 ± 2.54^a^	28.73 ± 5.32^a^	32.16 ± 1.84^a^	<0.001
Serum 25(OH)D levels, nmol/L	35.24 ± 7.52	40.57 ± 8.14	3.55 ± 2.25^a,b^	5.77 ± 2.07^a,b^	<0.001
Serum calcium levels, mmol/L	2.83 ± 0.22	2.86 ± 0.37	2.83 ± 0.36	3.15 ± 0.18	0.054

Data are presented as mean ± SD, *n* = 10 rats, ^a^*p* < 0.05 vs NR, ^b^*p* < 0.05 vs DC. [NR: nondiabetic rats with control diet; DC: diabetic rats with control diet; DD: diabetic rats with vitamin D-deficient diet; DDS: diabetic rats with vitamin D-deficient diet + calcitriol supplementation].

**Table 2:** Percentage of maximal relaxation (*R*_max_) to acetylcholine, sodium nitroprusside, and salbutamol and maximal contraction (*E*_max_) to calcium ionophore and phenylephrine in mesenteric arteries of experimental rats.

Groups		Endothelium-dependent responses		Smooth muscle responses		
		Acetylcholine	Calcium ionophore	Sodium nitroprusside	Salbutamol	Phenylephrine
NR	*R*_max_ (%) *E*_max_ (%)	74.56 (18.53)	27.19 (22.64)	69.30 (16.39)	91.12 (9.10)	118.50 (40.31)
DC	*R*_max_ (%) *E*_max_ (%)	53.48 (19.97)^a^	58.78 (25.59) ^a^	67.83 (22.11)	91.98 (6.58)	148.30 (63.77)
DD	*R*_max_ (%) *E*_max_ (%)	28.45 (10.70)^a,b^	97.55 (20.80)^a,b^	34.42 (9.44)^a,b^	86.28 (15.44)	141.50 (36.15)
DDS	*R*_max_ (%) *E*_max_ (%)	48.40 (8.41)^a,c^	90.67 (29.14)^a,b^	52.24 (16.04)	90.92 (17.34)	168.70 (72.90)

Data are presented as mean (SD), *n* = 10 rats, ^a^*p* < 0.05 vs NR, ^b^*p* < 0.05 vs DC, ^c^*p* < 0.05 vs DD [NR: nondiabetic rats with control diet; DC: diabetic rats with control diet; DD: diabetic rats with vitamin D-deficient diet; DDS: diabetic rats with vitamin D-deficient diet+calcitriol supplementation].

## Data Availability

The data used to support the findings of this study are included within the article.
